# Ultrasonic-Assisted Impregnation as an Efficient Tool for the Manufacture of Cu-Containing Faujasite as an Active Catalyst for the Oxidation of Cyclohexene

**DOI:** 10.1021/acsomega.5c01797

**Published:** 2025-06-16

**Authors:** Łukasz Kuterasiński, Agnieszka Wojtkiewicz, Grzegorz Kurowski, Piotr Jeleń, Maciej Sitarz, Małgorzata Ruggiero-Mikołajczyk, Mariusz Gackowski, Przemysław Jakub Jodłowski

**Affiliations:** † Jerzy Haber Institute of Catalysis and Surface Chemistry, Polish Academy of Sciences, Ul. Niezapominajek 8, Kraków 30-239, Poland; ‡ Faculty of Chemical Engineering and Technology, Cracow University of Technology, Ul. Warszawska 24, Kraków 31-155, Poland; § Faculty of Materials Science and Ceramics, 49811 AGH University of Krakow, Al. Adama Mickiewicza 30, Kraków 30-059, Poland

## Abstract

In this study, a method was developed to prepare active catalysts for cyclohexene oxidation by using ultrasonic-assisted impregnation of faujasite zeolites (in protonic or sodium forms) with copper. This reaction is important for producing valuable chemicals such as surfactants, polymers, agrochemicals, and pharmaceuticals. All catalysts were thoroughly characterized, mainly using spectroscopic techniques. The results showed that the chemical form of copper was influenced by the use of ultrasound. The effects were more pronounced when sodium-form zeolites were used and when the ultrasound treatment lasted longer. In these cases, copper tended to form clusters. Notably, the ultrasound treatment did not cause structural damage (amorphization) to the zeolite framework. Catalytic tests revealed that using ultrasound to prepare copper-loaded protonic faujasites significantly increased cyclohexene conversion from 1% to 13%, with a selectivity of 55% toward 2-cyclohexen-1-one. In contrast, for catalysts based on sodium-form zeolites, the conversion dropped sharply from 75% to 7%, while selectivity increased from 53% to 71%. This suggests that copper clusters formed during ultrasound treatment promote the formation of 2-cyclohexen-1-one.

## Graphical abstract

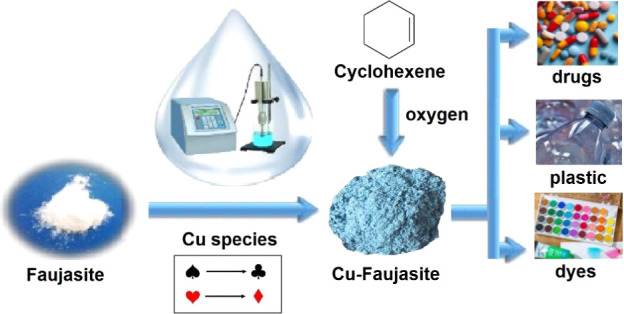

## 1 Introduction

One important area of scientific and technological research is the use of specially prepared materials as catalysts in various chemical processes. Sonication is a promising method for synthesizing advanced materials. This technique is often chosen because it enables unique reaction pathways that are not accessible through conventional methods, opening new possibilities for material development. One major advantage of using ultrasound (acoustic cavitation) in material synthesis and modification is the reduced synthesis time and energy consumption. It also eliminates the need for expensive or toxic reagents, ultimately lowering the overall production cost.^1,2^ Due to these benefits, the use of ultrasound in chemistry has gained increasing attention. Sonochemistry is now being explored as an alternative method in both laboratory and industrial applications, including medicine, catalysis, cosmetics, agriculture, food processing, construction materials, and materials engineering.^2–29^

Ultrasound has been applied in the synthesis of various zeolites, such as A,^4,5^ MCM-22,^6^ NaP,^7^ CHA,^8,9^ T,^10^ FAU,^11–13^ MFI,^14^ and BEA.^14^ These materials showed improved crystallinity and smaller crystal sizes. Ultrasound has also been used for postsynthesis modifications of zeolites, often with better results than traditional methods. For example, Hosseini et al.^15^ used ultrasound to assist in the dealumination of zeolite Y using ethanol-acetylacetone as a chelating agent. Other researchers^16–20^ studied mesoporous zeolites prepared with ultrasound, which showed higher mesoporosity and better catalytic performance compared to samples modified without ultrasound.

Another application of ultrasound is in depositing active metal species onto zeolite supports. Studies^21,22^ showed that sonochemical methods improved the dispersion of copper on zeolites. For instance, copper-loaded ZSM-5 and USY zeolites prepared with ultrasound achieved nearly 100% selectivity in the selective catalytic reduction of NO_*x*_. Similarly, Cu-containing BEA zeolites prepared sonically showed excellent performance in converting lactic acid to acrylic acid.^22^

Understanding the state of copper within the zeolite structure is essential for explaining its catalytic behavior. Copper can be introduced into zeolites through ion exchange or impregnation methods,^23^ and it can exist in oxidation states +2, + 1, or 0. Inside zeolites, copper is typically found as Cu^+^ or Cu^2+^ ions, either in exchange positions or as oxide clusters.^23–36^ The location of copper has been studied in various zeolite frameworks, including BEA,^23,24^ MFI,^25–29^ MOR,^24,30^ LTA,^24,31^ CHA,^24,32^ and FAU.^24,25,33–36^

Cu^+^ ions can be introduced by ion exchange, by reducing Cu^2+^ with CO, or through autoreduction of Cu^2+^-zeolites. Autoreduction involves heating in a vacuum or inert atmosphere, converting Cu^2+^ to Cu^+^ while oxidizing the zeolite framework: Cu^2+^-Z→ Cu^+^-Z^+^, where Z and Z^+^ represent the zeolite framework and its oxidized form. Alternatively, extraframework ligands may participate in the reduction, as shown in the following reactions:^37^

1 2[CuOH]+→[Cu−O−Cu]2++H2O→2Cu++12O2

2 2[CuOH]+→Cu++Cu2+−O−+H2O→2Cu++12O2

Nachtigall and Nachtigallova^38,39^ modeled Cu^+^ in MFI zeolites and found that it coordinates with two oxygen atoms from one [AlO_4_]^−^ tetrahedron, often located in six-membered rings (6MRs) of the zeolite channels. The reverse of autoreduction can regenerate Cu^2+^ species by reacting Cu^+^ with O_2_. In low Si/Al zeolites, this can lead to incorporation of O_2_^–^ into the framework or formation of oxo-complexes, depending on the reaction pathway.^40^

Cyclohexene oxidation is a key reaction in organic chemistry, producing intermediates for drugs, surfactants, agrochemicals, and polymers.^41^ However, the reaction mechanism is complex due to the challenge of activating C–H bonds and forming C–O bonds, while both allylic C–H and C=C bonds are easily oxidized. This often results in low selectivity and yields.^42,43^ Despite this, the reaction remains important because the size of the cyclohexene molecule is similar to many chemical intermediates.^44^

This study focuses on developing an ultrasonic-assisted method for impregnating faujasite-type zeolites (Si/Al = 31) with copper to create efficient catalysts for cyclohexene oxidation. The catalysts were thoroughly characterized using in situ spectroscopic techniques. The research also examined how ultrasound conditions affect the chemical form and distribution of copper species, and how these factors influence the physicochemical and catalytic properties of the resulting materials. Notably, no previous studies were found on using sonochemically prepared Cu-zeolites for cyclohexene oxidation.

## 2 Experimental Section

### 2.1 Sample Preparation

A commercial FAU-type zeolite with a Si/Al ratio of 31 (CBV 760) was obtained from Zeolyst Company (Farmsum, The Netherlands). The Si/Al ratio was confirmed using ICP-OES analysis.^20^ This zeolite, originally in the protonic form (referred to as HF31), was also converted into its sodium form (NaF31) by performing five consecutive ion exchanges with a 0.5 M aqueous sodium nitrate solution at 80 °C for 2 h. The solution-to-zeolite mass ratio was 30:1. After ion exchange, the NaF31 sample was centrifuged at 4000 rpm, dried overnight at 60 °C, and calcined at 500 °C for 3 h.

Both HF31 and NaF31 zeolites were used as supports for copper loading. Copper was introduced via wet impregnation, either with or without ultrasonic assistance. The copper content was set at 2 wt % for HF31 and 5 wt % for NaF31. For each 2 g of zeolite, 200 mL of an aqueous Cu­(NO_3_)_2_·3H_2_O solution was used, containing either 0.150 g (for HF31) or 0.375 g (for NaF31) of the copper salt. After impregnation, the samples were dried overnight at 80 °C and calcined at 500 °C for 3 h. These specific copper loadings were chosen based on previous findings showing that they lead to the most noticeable differences in the chemical form of copper introduced into faujasite supports under standard conditions.^35,36^

Ultrasonic-assisted impregnation was carried out using a QSonica Q700 sonicator (20 kHz, 60 W) with a 1/2-in. diameter horn (Church Hill Rd, Newtown, CT, USA). The sonication times were 0 min (no ultrasound), 15, 30, and 60 min. Depending on the zeolite type (HF31 or NaF31) and the sonication time, the resulting samples were labeled as follows: CuHF31, CuHF31 (15), CuHF31 (30), and CuHF31 (60) vs CuNaF31, CuNaF31 (15), CuNaF31 (30), and CuNaF31 (60), respectively.

### 2.2 Sample Characterization

The crystallinity of the prepared samples was analyzed using X-ray diffraction (XRD) with a PANalytical X’Pert PRO MPD diffractometer (40 kV, 30 mA) equipped with a CuKα radiation source (λ = 1.5418 Å). Measurements were taken over a 2θ range of 5–50° with a step size of 0.033°.

The silicon environment in the samples was studied using solid-state ^29^Si MAS NMR spectroscopy on a Bruker Advance III 500 MHz spectrometer (11.7 T, 99.4 MHz) with an 8 kHz spinning rate. Measurements were performed in zirconia rotors with high-power proton decoupling (SPINAL64), using 5.8 μs (π/3) pulses and a 20 s repetition time. Chemical shifts were referenced to tetramethylsilane (TMS; >99%).

Porosity was evaluated by nitrogen adsorption at −196 °C using an Autosorb-1 Quantachrome analyzer. Brunauer–Emmett–Teller (BET), the Barrett–Joyner–Halenda (BJH), and the t-plot methods were applied. Prior to measurements, samples were degassed under vacuum at 250 °C overnight.

Sample morphology was examined using scanning electron microscopy (SEM) with an FEI Nova Nano SEM 200 in backscattered electron mode. Elemental mapping (SEM/EDS) was performed with a JEOL 5400 microscope and a LINK ISIS microprobe analyzer. Samples were coated with a thin carbon layer before analysis.

FT-IR spectra were recorded using a NICOLET iS10 spectrometer (Thermo Scientific) with an MCT detector, in the range of 4000–650 cm^–1^, at 4 cm^–1^ resolution and 128 scans per spectrum. Samples (ca. 70 mg) were pressed into self-supporting wafers and activated under vacuum at 400 °C for 1 h before measurement. CO (Air Products, 99.95%) adsorption studies were used to identify and quantify copper species. Bands at 2160 cm^–1^ correspond to Cu^+^ in exchange positions (Cu^+^ exch, ε = 1.3 cm^2^/μmol), while bands at 2137–2140 cm^–1^ indicate Cu^+^ in oxide form (Cu^+^ ox, ε = 0.91 cm^2^/μmol).

The degree of Na^+^/H^+^ ion exchange in HF31 and NaF31 was assessed by IR spectroscopy of ammonia adsorption at 120 °C, based on the intensity of the 1450 cm^–1^ band (NH_3_ on Brønsted acid sites). Similarly, Cu^+^/H^+^ exchange was evaluated by comparing this band across HF31, CuF31, and CuF31 (60) samples.

Diffuse Reflectance UV–vis (DR-UV–vis) spectra were collected using an AvaSpec-ULS3648 spectrometer with a high-temperature reflection probe and a Praying Mantis reaction chamber. The light source was a deuterium-halogen lamp (AvaLight-D­(H)-S), and spectra were recorded from 200 to 500 nm using AvaSoft v9.0 software. Samples were dehydrated at 110 °C under helium flow (30 mL/min) before analysis.

Cyclohexene oxidation was used as a test reaction to evaluate catalytic performance. Reactions were carried out in a Buchi Miniclave stainless steel reactor at 80 °C under 10 bar of O_2_ for 6 h, using 50 mg of catalyst and 10 mL of cyclohexene. The reactor was purged with O_2_ for 15 min before each run. After the reaction, the mixture was cooled in an ice bath and treated with 10 mg of triphenylphosphine (PPh_3_) to prevent further oxidation. Products were analyzed using a Thermo Scientific Trace 1310 gas chromatograph coupled with a single quadrupole mass spectrometer (ISQ) and an RXi-5MS capillary column (30 m, 0.25 mm ID, 0.25 μm film thickness).

## 3 Results and Discussion

### 3.1 Crystallinity, Porosity, and Morphology

The crystallinity of the catalysts was evaluated using X-ray diffraction (XRD). As shown in Figure S1, all samples exhibited diffraction patterns characteristic of the faujasite structure.^45^ The method of catalyst preparation did not affect the XRD reflections. This finding is supported by the ^29^Si MAS NMR spectra (Figure S2), which showed no increase in the broad signal at −112 ppm, typically associated with amorphous Si­(0Al) species.^46–48^ Therefore, no amorphization occurred, regardless of the preparation method. No additional XRD peaks related to copper phases were detected in any of the Cu-faujasite samples (both H- and Na-series). This may be due to the formation of copper species too small or too dispersed to be detected by XRD.

Porosity data are presented in Table 1 and Figure S3. The nitrogen adsorption–desorption isotherms were classified as type IV with H4 hysteresis loops, indicating mesoporous characteristics.^49^ However, the hysteresis is likely due to intercrystalline voids between faujasite crystals, which were also observed in other microporous zeolites, as reported by Fajula et al.,^50^ Rac et al.,^51^ Hasan et al.,^52^ Verboekend et al.,^53^ Li et al.,^54^ and Silaghi et al.^55^ Quantitative analysis (Table 1) shows that the preparation method had only a minor effect on the porous structure. Samples based on sodium-form zeolites had slightly lower surface areas and pore volumes compared to those based on protonic zeolites. In both series, copper impregnation led to a slight decrease in specific surface area, total pore volume, and micropore volume, while the average pore diameter remained nearly unchanged. The influence of ultrasound during copper impregnation on porosity was not consistent and varied depending on the sample.

**Table 1:** Porosity of the Sonochemically Prepared Cu-Containing F31 Samples

	Porous structure			
Sample	V_micro_ [cm^3^/g]	V_total_ [cm^3^/g]	S_BET_ [m^2^/g]	D [Å]
HF31	0.324	0.570	915	25.0
CuHF31	0.239	0.635	840	30.2
CuHF31 (15)	0.235	0.562	827	27.2
CuHF31 (30)	0.288	0.532	811	26.2
CuHF31 (60)	0.302	0.633	937	27.0
NaF31	0.204	0.497	762	26.1
CuNaF31	0.179	0.443	647	27.4
CuNaF31 (15)	0.178	0.495	657	30.2
CuNaF31 (30)	0.187	0.494	685	28.9
CuNaF31 (60)	0.173	0.468	611	30.6

SEM images of the modified samples are shown in Figures S4 and S5. All catalysts exhibited irregularly shaped particles with sizes up to 1 μm. In each case, well-defined prismatic grains with sharp edges were observed. Neither the type of faujasite support (HF31 vs NaF31) nor the use of ultrasound during copper impregnation had any noticeable effect on the morphology of the materials. Importantly, no amorphous phases were detected in any of the Cu-impregnated protonic or sodium faujasite samples. The preserved morphology of the Cu-modified zeolites is consistent with their maintained crystallinity and the minimal changes observed in porosity.

### 3.2 Distribution and Chemical Structure of Copper Active Phase

The distribution of copper on the faujasite supports was examined using energy-dispersive X-ray spectroscopy (EDS) mapping, as shown in Figures 1, S6, and S7. Figure 1 highlights the effect of the zeolite type (HF31 vs NaF31) on copper distribution after 60 min of ultrasonic treatment during impregnation. The EDS maps revealed that the type of zeolite support significantly influenced copper dispersion. In the CuHF31 (60) sample, based on protonic faujasite, copper was evenly distributed. In contrast, the CuNaF31 (60) sample, based on sodium faujasite, showed visible copper clusters appearing as bright spots. Copper’s tendency to form clusters is well documented in the literature.^23–36^ This difference in copper distribution and chemical form is attributed to distinct reaction pathways during impregnation. In the case of CuHF31 (60), ultrasonic treatment of protonic faujasite with Cu­(NO_3_)_2_ led to the formation of HNO_3_, which was removed during calcination. This removal shifted the ion exchange equilibrium toward the formation of CuHFAU. However, for the sodium form (NaF31), ultrasonic impregnation with Cu­(NO_3_)_2_ produced NaNO_3_, which could not be removed by calcination. As a result, less copper entered exchange positions, and the remaining Cu^+^ and Cu^2+^ formed oxide clusters. These findings are consistent with previous studies.^35,36^

**Figure 1:**
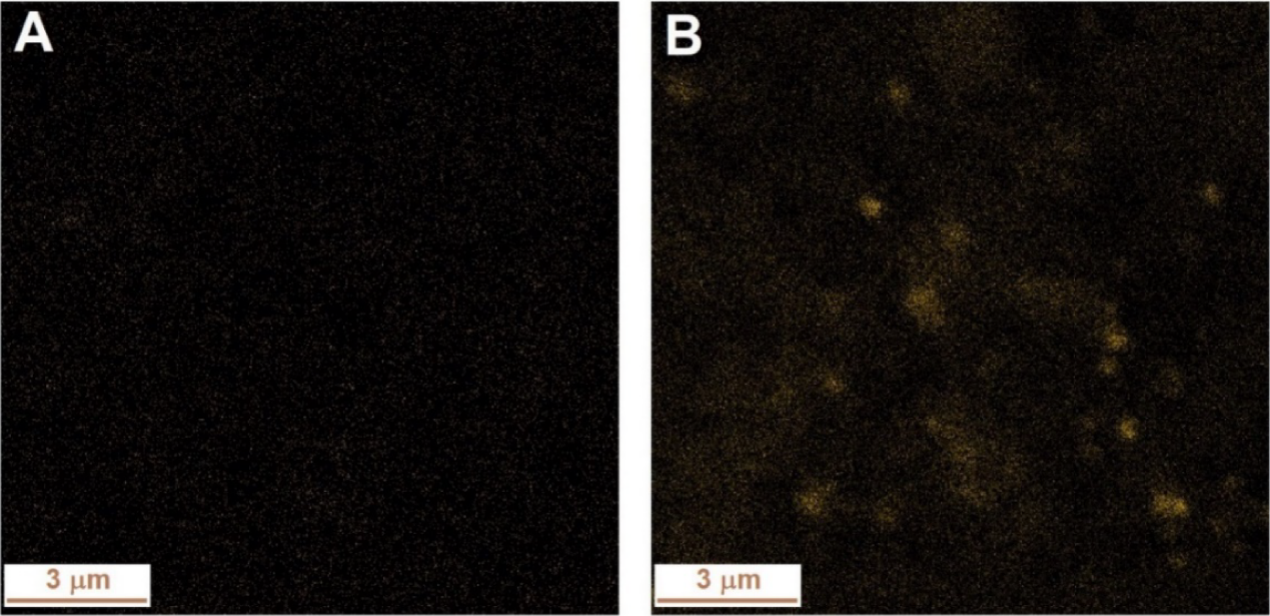
Influence of carrier provenance (HF31 vs NaF31) on the appearance of Energy-dispersive X-ray spectroscopy (EDS) distribution maps over the surface of Cu-containing zeolite samples prepared sonochemically for 60 min. (A) CuHF31(60) and (B) CuNaF31(60) samples.

An important question addressed in this study was how the duration of ultrasonic-assisted copper deposition affects the distribution and chemical form of copper species, and how this effect varies depending on the type of zeolite support (HF31 vs NaF31). Based on the comparative analysis of EDS maps (Figures S6 and S7), it was found that for the HF31-based samples (Figure S6), neither the distribution nor the chemical form of copper appeared to change significantly with increasing ultrasound exposure time. In contrast, the NaF31-based samples (Figure S7) showed a different trend. In these samples, longer ultrasound exposure led to more pronounced copper clustering on the zeolite surface. This suggests that the sodium form of faujasite is more prone to copper aggregation under ultrasonic conditions.

The qualitative and quantitative characterization of copper species is presented in Figure 2 and Table S1. All Cu-containing samples showed two characteristic FT-IR bands: a sharp peak at 2160 cm^–1^ corresponding to exchangeable Cu^+^ ions (Cu^+^_exch_) bonded with CO, and a broader band in the 2137–2145 cm^–1^ range, attributed to CO interacting with Cu^+^ in oxide form (Cu^+^_oxide_).^25^

**Figure 2:**
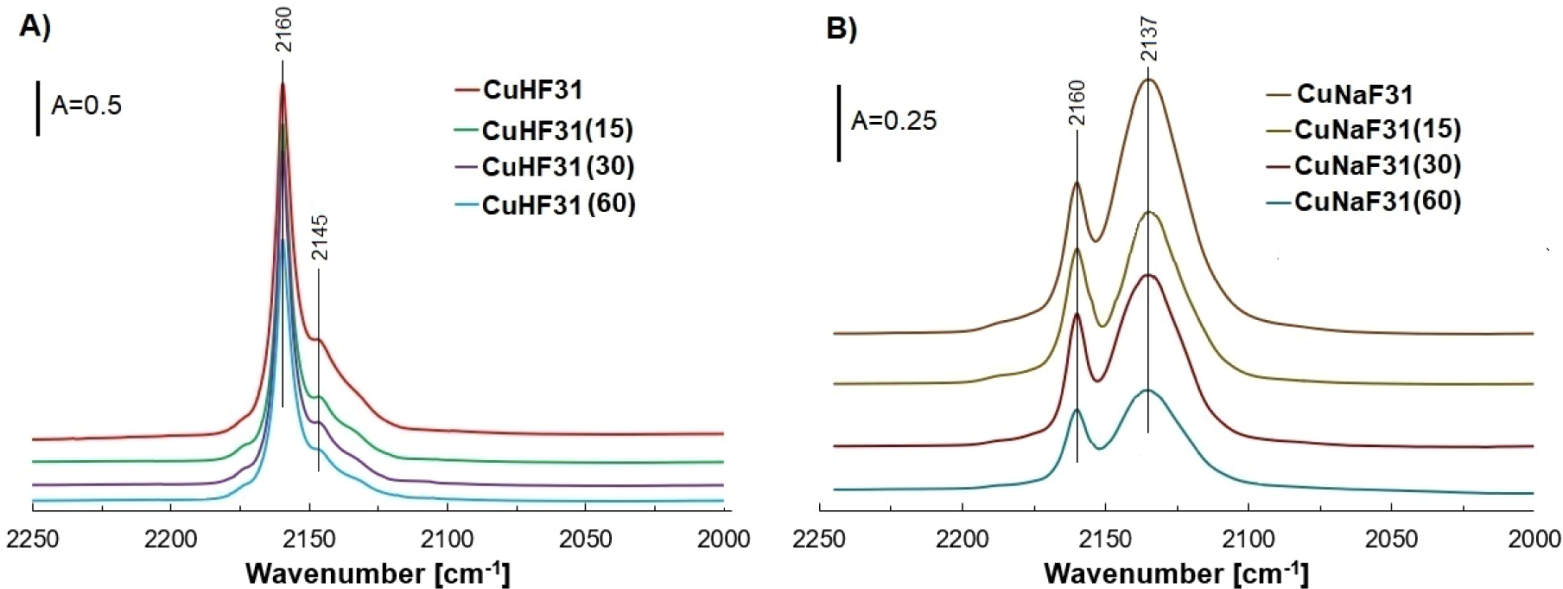
FT-IR spectra illustrating the adsorption of CO over various copper species in Cu–F31 samples based on (A) HF31 and (B) NaF31 carriers.

From the combined analysis of Figure 2 and Table S1, it can be concluded that both the type of zeolite support and the duration of ultrasonic treatment influenced the chemical form and distribution of copper species. In the HF31-based samples, increasing the ultrasound exposure time from 0 to 60 min led to a gradual decrease in the intensity of both Cu^+^_exch_ and Cu^+^_oxide_ bands, indicating a reduction in the concentration of these species.

The FT-IR spectra of Cu-zeolites based on sodium faujasite (Figure 2B) showed a different pattern compared to those based on protonic zeolite. Notably, the band at ∼2137 cm^–1^ (Cu^+^_oxide_) was more intense than the 2160 cm^–1^ band (Cu^+^_exch_), which aligns with the quantitative data in Table S1, indicating a higher concentration of Cu^+^_oxide_ species. This suggests that in Na-zeolite samples, the following equilibrium is dominant: Na-zeolite + copper­(II) nitrate → Cu-zeolite + sodium nitrate. Since NaNO_3_ cannot be removed by calcination, unreacted Cu­(NO_3_)_2_ decomposes into copper oxide species, which tend to form clusters. These findings are consistent with the EDS analysis and previously reported studies.^35,36^

The effect of ultrasound on Cu-zeolites based on Na-faujasite was similar to that observed for the protonic series. Sonication reduced the intensity of FT-IR bands corresponding to both Cu^+^_exch_ and Cu^+^_oxide_ species (Figure 2B).

In both series (HF31 and NaF31), ultrasound treatment led to a noticeable decrease in the accessibility of copper species to the CO probe molecule. A comparison between the total copper content and the concentrations determined by CO sorption (Table S1) revealed that much of the copper was not accessible to CO. This could be due to copper being located in inaccessible sites or forming large agglomerates.^33^

EDS analysis (Table S1) showed that the measured copper content was higher than the amount introduced via impregnation, suggesting that most copper was located on the external surface of the zeolite grains. Interestingly, ultrasound had no effect on surface copper content in the H-series, while in the Na-series, it caused a slight decrease, possibly reducing EDS detectability. The weak correlation between copper content and ultrasound duration (especially in the H-series) suggests that copper did not migrate into the zeolite interior. Instead, the decreasing FT-IR band intensities likely result from the formation of copper clusters, which are difficult to detect by EDS.

Table S1 also shows that the Na^+^/H^+^ ion exchange in Na-faujasite was highly efficient (>90%). In contrast, the Cu^+^/H^+^ exchange during copper impregnation of HF31 was lower (77%) and decreased further with ultrasound treatment (down to 70%).

To better understand the chemical form of copper introduced into the zeolites, diffuse reflectance UV–vis (DR-UV–vis) spectroscopy was used (Figure 3). Unlike FT-IR, which probes surface-accessible species, DR-UV–vis provides information about copper species throughout the entire zeolite grain.

**Figure 3:**
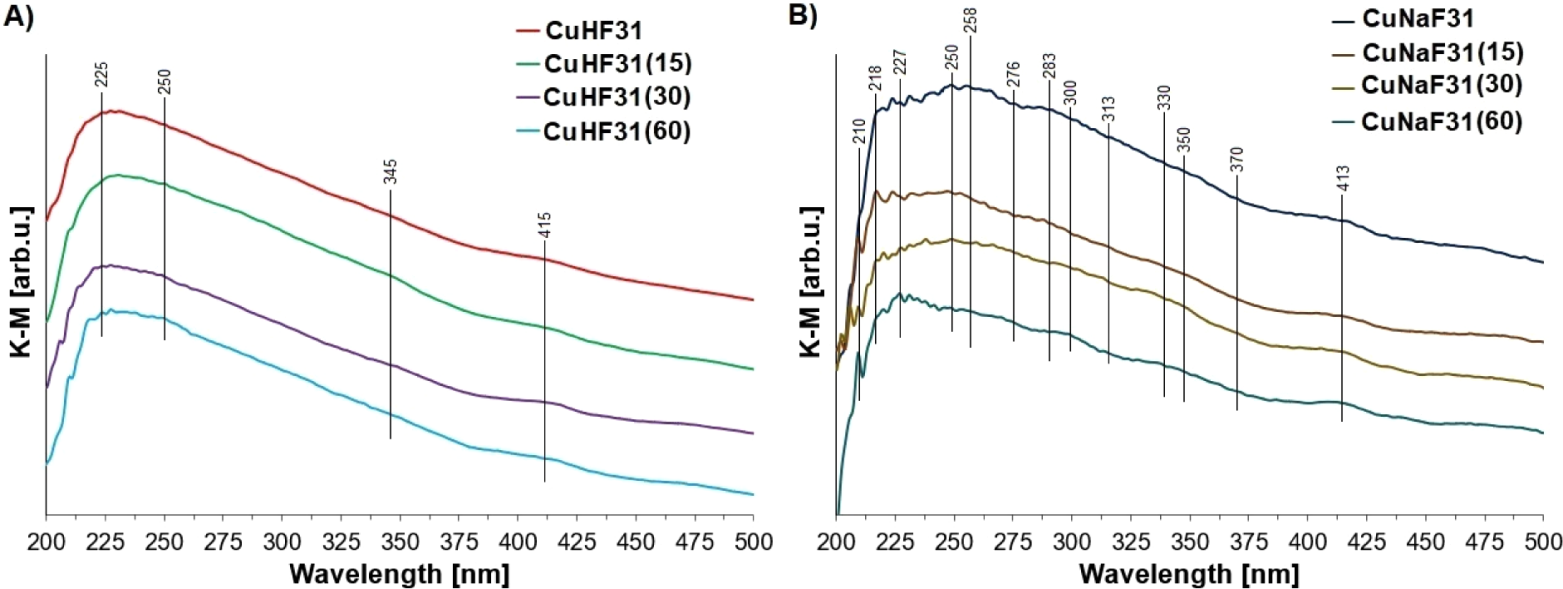
In situ diffuse reflectance UV/visible (UV/vis) spectra of prepared catalysts in different times of the application of ultrasonic irradiation in the Cu deposition for (A) HF31 and (B) NaF31 carriers.

The UV–vis spectra revealed that the type of zeolite support influenced the form of copper. In HF31-based samples, broad absorption bands were observed at 225, 250, 345, and 415 nm (Figure 3A). The bands at 225 and 250 nm are likely due to Cu^+^_exch_ species,^56^ while the weak band at 345 nm is attributed to [Cu–O–Cu]_n_ clusters.^57^ The broad band at 415 nm corresponds to Cu_2_O_2_-type species, such as bis­(μ-oxo)­dicopper and peroxo-dicopper complexes, as described by Groothaert et al.^58^

Ultrasonic-assisted impregnation, especially for 30 and 60 min (CuHF31 (30) and CuHF31 (60)), slightly increased the intensity of the 415 nm band, suggesting a minor transformation of Cu^+^_exch_ into dicopper dioxide species.

The DR-UV–vis spectra of catalysts based on sodium faujasite (Figure 3B) showed a more complex pattern, indicating a broader variety of monovalent and divalent copper species in both exchangeable and oxide forms. In these spectra, three main absorption regions can be identified: λ < 260 nm attributed to Cu^+^ species dispersed on the surface of the materials,^56^ 270 nm < λ < 330 nm associated with charge transfer between monomeric Cu^2+^ ions and oxygen atoms,^59^ and λ > 330 nm corresponding to the presence of Cu_*x*_O_*y*_-type copper oxide clusters.^60^ Similar to the protonic faujasite-based samples, ultrasonic treatment of Na-faujasite with aqueous Cu­(NO_3_)_2_ led to a noticeable increase in the intensity of the band at 413 nm, which is characteristic of Cu_2_O_2_ species. These UV–vis results fully support the conclusions drawn from FT-IR and EDS analyses, confirming that both the type of zeolite support (HF31 vs NaF31) and the duration of ultrasonic treatment significantly influence the chemical form of the copper active phase.

### 3.3 Catalytic Properties

Selected samples were tested as catalysts in the oxidation of cyclohexene. The catalysts included Cu-loaded protonic and sodium faujasites, prepared either by conventional impregnation or by ultrasonic-assisted impregnation for 60 min. The catalytic results are summarized in Table 2.

**Table 2:** Catalytic Properties of Variously Prepared Cu–F31 Samples in the Oxidation of Cyclohexene

		Selectivity [%]			
Sample	Conversion [%]	2-Cyclohexenol	2-Cyclohexenone	Bi-2-cyclohexen-1-yl	2-Cyclohexene-1,4-diol
CuHF31	1	n.a.	n.a.	n.a.	n.a.
CuHF31(60)	13	27	55	7	11
CuNaF31	75	39	53	1	5
CuNaF31(60)	7	19	71	3	7

The catalyst CuHF31, prepared without ultrasound, showed very low activity, with only 1% cyclohexene conversion, too low to meaningfully discuss product selectivity. However, the sonochemically treated counterpart, CuHF31(60), showed a significant improvement, reaching 13% conversion. The selectivity to 2-cyclohexen-1-ol, 2-cyclohexen-1-one, Bi-2-cyclohexen-1-yl, and 2-cyclohexene-1,4-diol was 27%, 55%, 7%, and 11%, respectively.

In contrast, the sodium-based catalyst CuNaF31 (prepared without ultrasound) exhibited high activity, achieving 75% conversion with a selectivity to 2-cyclohexen-1-ol, 2-cyclohexen-1-one, Bi-2-cyclohexen-1-yl, and 2-cyclohexene-1,4-diol of 39%, 53%, 1%, and 5%, respectively. For this sample, the trace selectivity to 4-hydroxy-2-cyclohexen-1-one was found (2%). However, when ultrasound was applied during preparation (CuNaF31(60)), the catalytic performance dropped significantly. Cyclohexene conversion decreased nearly 10-fold—from 75% to 7%. Selectivity to 2-cyclohexen-1-ol also dropped from 39% to 19%. On the other hand, selectivity to 2-cyclohexen-1-one increased from 53% to 71%, with slight increases in Bi-2-cyclohexen-1-yl (from 1% to 3%) and 2-cyclohexene-1,4-diol (from 5% to 7%).

A cross-analysis of the catalytic data shows that the effect of ultrasound strongly depends on the chemical form of the zeolite support. When protonic faujasite was used, ultrasonic treatment enabled the formation of an active Cu-zeolite catalyst for cyclohexene oxidation. In contrast, sodium faujasite suppressed catalytic activity. However, ultrasound consistently promoted the formation of 2-cyclohexen-1-one, regardless of the support type.

Comparing catalytic performance with the distribution and chemical form of copper species (from EDS and FT-IR data) suggests that copper clusters reduce overall cyclohexene conversion and the formation of 2-cyclohexen-1-ol, but enhance selectivity toward 2-cyclohexen-1-one. It is important to note that only the copper species accessible to reactants contribute to catalytic activity. Thus, the rest of the copper should not influence the catalytic properties of the studied samples.

Comparing these results with literature is challenging due to limited studies on Cu-zeolites for cyclohexene oxidation. However, Maryam et al.^61^ reported 81% conversion and 65% selectivity to 2-cyclohexen-1-one using a Cu­(II)-Schiff base complex encapsulated in faujasite. Godhani et al.^62^ achieved 86% conversion with 40% and 60% selectivity to 2-cyclohexen-1-ol and 2-cyclohexen-1-one, respectively, using ligand-supported Cu on zeolite Y.

Other copper-based catalysts on nonzeolite supports have also been studied. Denekamp et al.^63^ used nanometric CuO on N-doped porous carbon (Cu/N:C), achieving 70–80% conversion and 40–50% selectivity to 2-cyclohexen-1-one. Cancino et al.^64^ investigated Cu-MOF catalysts in different solvents. In water–dichloroethane, conversion ranged from 16–34% with 72–77% selectivity to 2-cyclohexen-1-one. In *n*-decane, conversion dropped to 19–24% and selectivity to 64–70%.

## 4 Summary and Conclusions

This study investigated the influence of ultrasonic irradiation on the chemical form and distribution of copper species supported on faujasite-type zeolites (Si/Al = 31) in both protonic and sodium forms, as well as its impact on catalytic performance in the oxidation of cyclohexene.

Based on FT-IR-monitored CO sorption, EDS mapping, and DR-UV–vis spectroscopy, it was found that copper species located on the external surface of the zeolites were highly sensitive to ultrasound. This effect strongly depended on the type of zeolite support and the duration of ultrasonic treatment. More pronounced changes were observed for sodium-form zeolites and longer sonication times, where copper tended to form clusters. Importantly, ultrasonic-assisted impregnation did not lead to amorphization of the zeolite structure.

Catalytic testing confirmed that both the zeolite type and the surface chemical form of copper significantly influenced catalytic behavior. For catalysts based on protonic faujasite treated with ultrasound for 60 min, cyclohexene conversion increased from 1% to 13%, with 55% selectivity toward 2-cyclohexen-1-one. In contrast, the sodium-based catalyst showed a sharp decrease in conversion from 75% to 7% after ultrasonic treatment, while selectivity to 2-cyclohexen-1-one increased from 53% to 71%.

These results suggest that the formation of copper clusters, promoted by ultrasound, particularly in sodium-form zeolites, suppresses overall conversion but enhances the selective formation of 2-cyclohexen-1-one.

## Supplementary Material



## Notes

Supplementary results on PXRD patterns, ^29^Si MAS NMR spectra, adsorption–desorption isotherms of nitrogen at −196 °C, SEM images, EDS distribution maps, and a table illustrating the chemical composition of variously prepared Cu–F31 samples (PDF)
