# Supplementary material for: Ultrasonic-Assisted Impregnation as an Efficient Tool for the Manufacture of Cu-Containing Faujasite as an Active Catalyst for the Oxidation of Cyclohexene
Source: ACS Omega. 2025 Jun 16;10(25):26884–91. doi: 10.1021/acsomega.5c01797 (PMC12223850; doi:10.1021/acsomega.5c01797)
Supplement: Supplementary file 1 [file ao5c01797_si_001.pdf]

# SUPPORTING INFORMATION

## Ultrasonic-assisted impregnation as an efficient tool for the manufacture of Cu-containing faujasite as an active catalyst for the oxidation of cyclohexene

Łukasz Kuterasiński[1*], Agnieszka Wojtkiewicz[1], Grzegorz Kurowski[2], Piotr Jeleń[3], Maciej Sitarz[3], Małgorzata Ruggiero-Mikołajczyk[1], Mariusz Gackowski[1], Przemysław Jakub Jodłowski[2]

*[1]Jerzy Haber Institute of Catalysis and Surface Chemistry, Polish Academy of Sciences, ul. Niezapominajek 8, 30-239 Krakow, Poland*
*[2]Faculty of Chemical Engineering and Technology, Krakow University of Technology, ul. Warszawska 24, 31-155 Krakow, Poland*
*[3] Faculty of Materials Science and Ceramics, AGH University of Krakow, Al. Mickiewicza 30, 30-059 Krakow, Poland*

**Corresponding author: e-mail: lukasz.kuterasinski@ikifp.edu.pl*

# Results

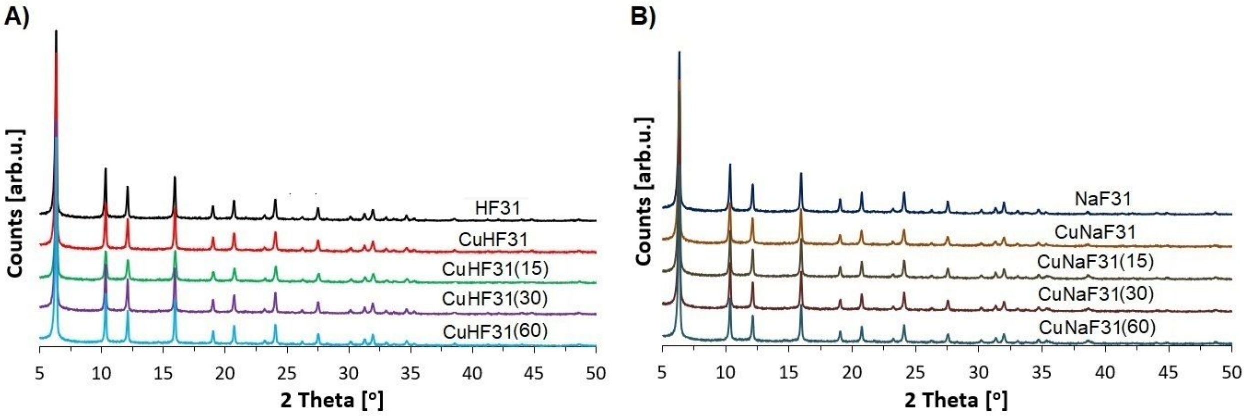


**Figure S1**. Powder XRD patterns of Cu-containing FAU-type zeolites for **(A)** HF31 and **(B)** NaF31 carriers.

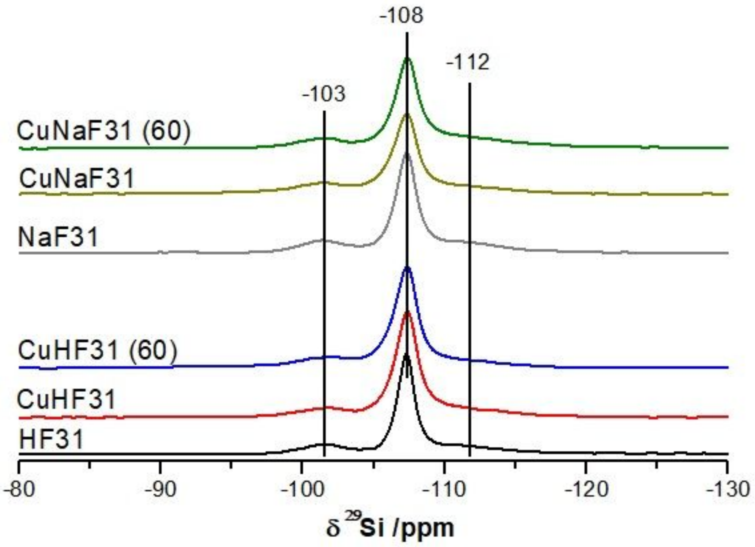


**Figure S2.** $^{29}$Si MAS NMR spectra of the studied samples at different times of the application of ultrasonic irradiation in the Cu deposition for HF31 and NaF31 carriers.

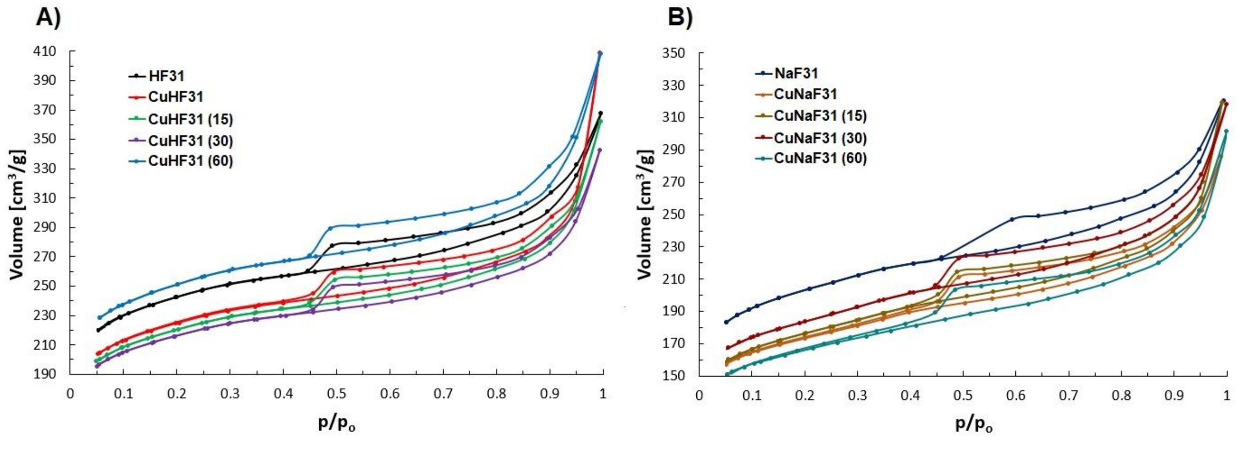


**Figure S3.** Adsorption-desorption isotherms of the nitrogen at -196 $^{o}$C for the studied samples belonging to **(A)** HF31 and **(B)** NaF31 carriers.

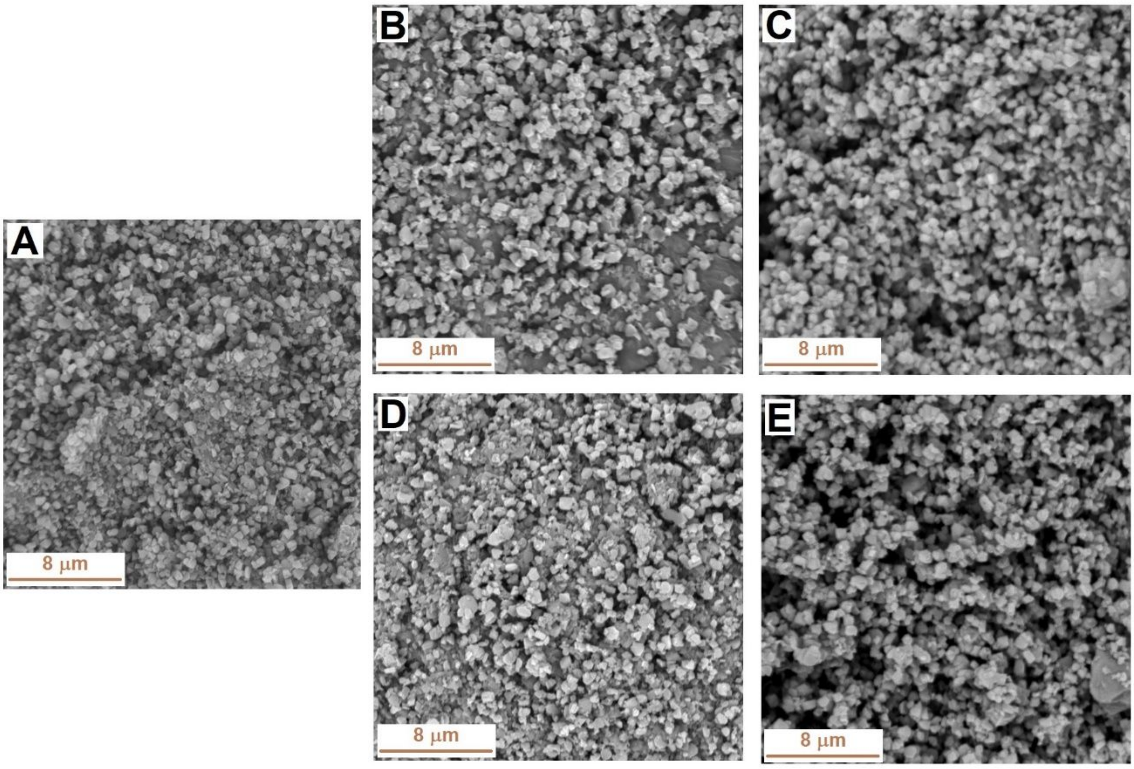


**Figure S4.** The impact of the duration of ultrasonic-assisted impregnation of Cu over HFAU31 zeolite on the morphology of the prepared samples. **(A)** HF31, **(B)** CuHF31, **(C)** CuHF31 (15), **(D)** CuHF31 (30), and **(E)** CuHF31 (60).

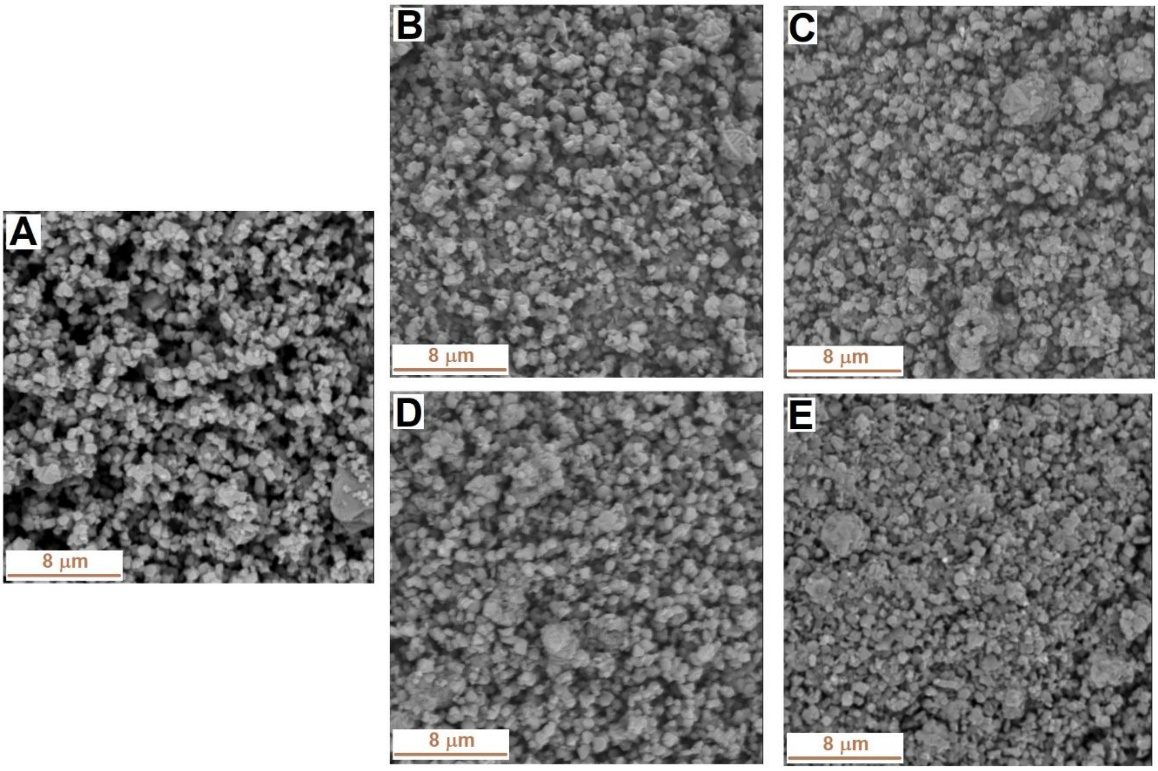


**Figure S5.** The impact of the duration of ultrasonic-assisted impregnation of Cu over NaFAU31 zeolite on the morphology of the prepared samples. **(A)** NaF31, **(B)** CuNaF31, **(C)** CuNaF31 (15), **(D)** CuNaF31 (30), and **(E)** CuNaF31 (60).

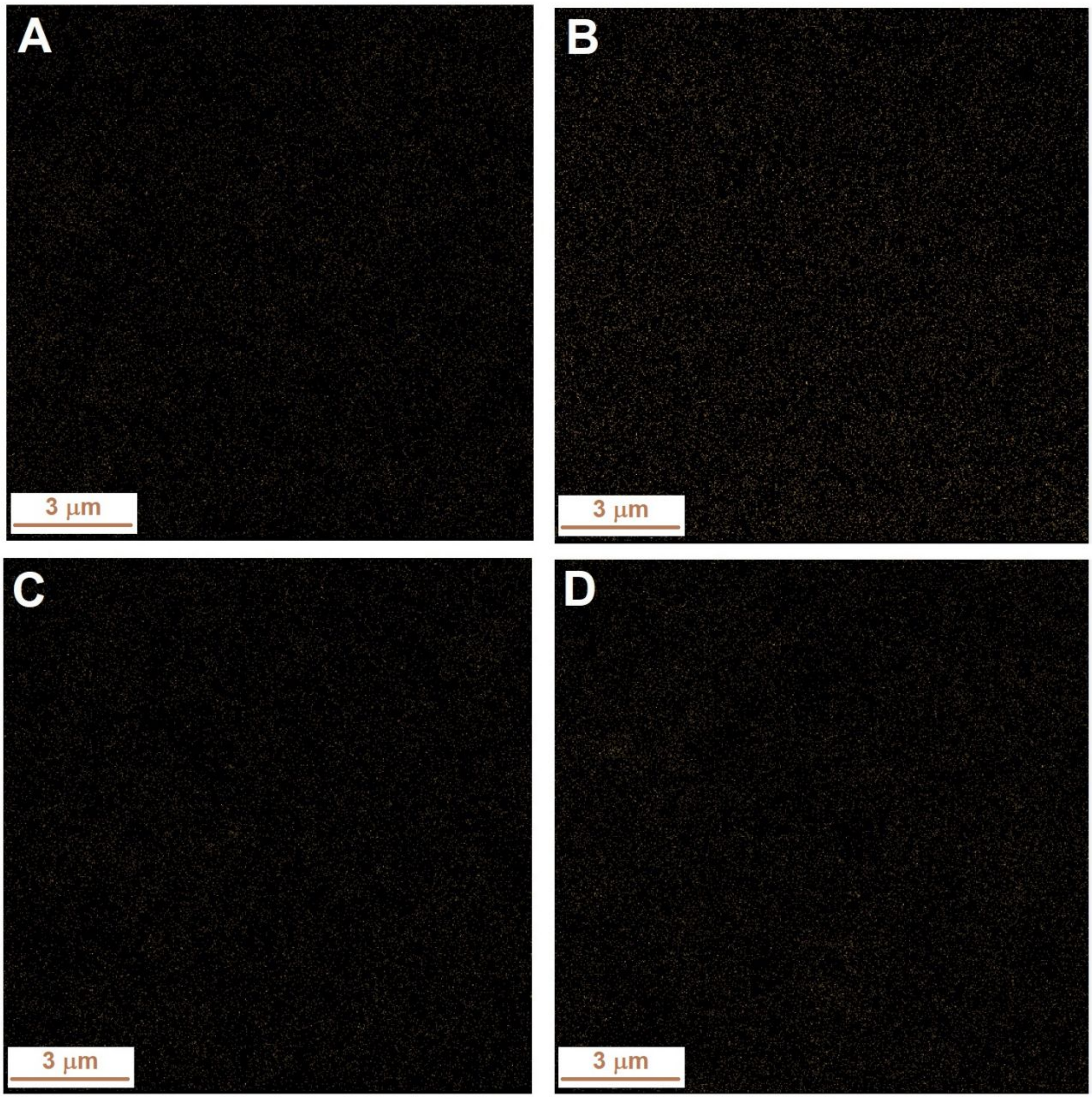


**Figure S6.** The influence of the duration of the ultrasonic-assisted Cu deposition on the appearance of Energy-dispersive X-ray spectroscopy (EDS) distribution maps over the surface of Cu-containing zeolite samples from the HF31 series. **(A)** CuHF31, **(B)** CuHF31 (15), **(C)** CuHF31 (30), and **(D)** CuHF31 (60).

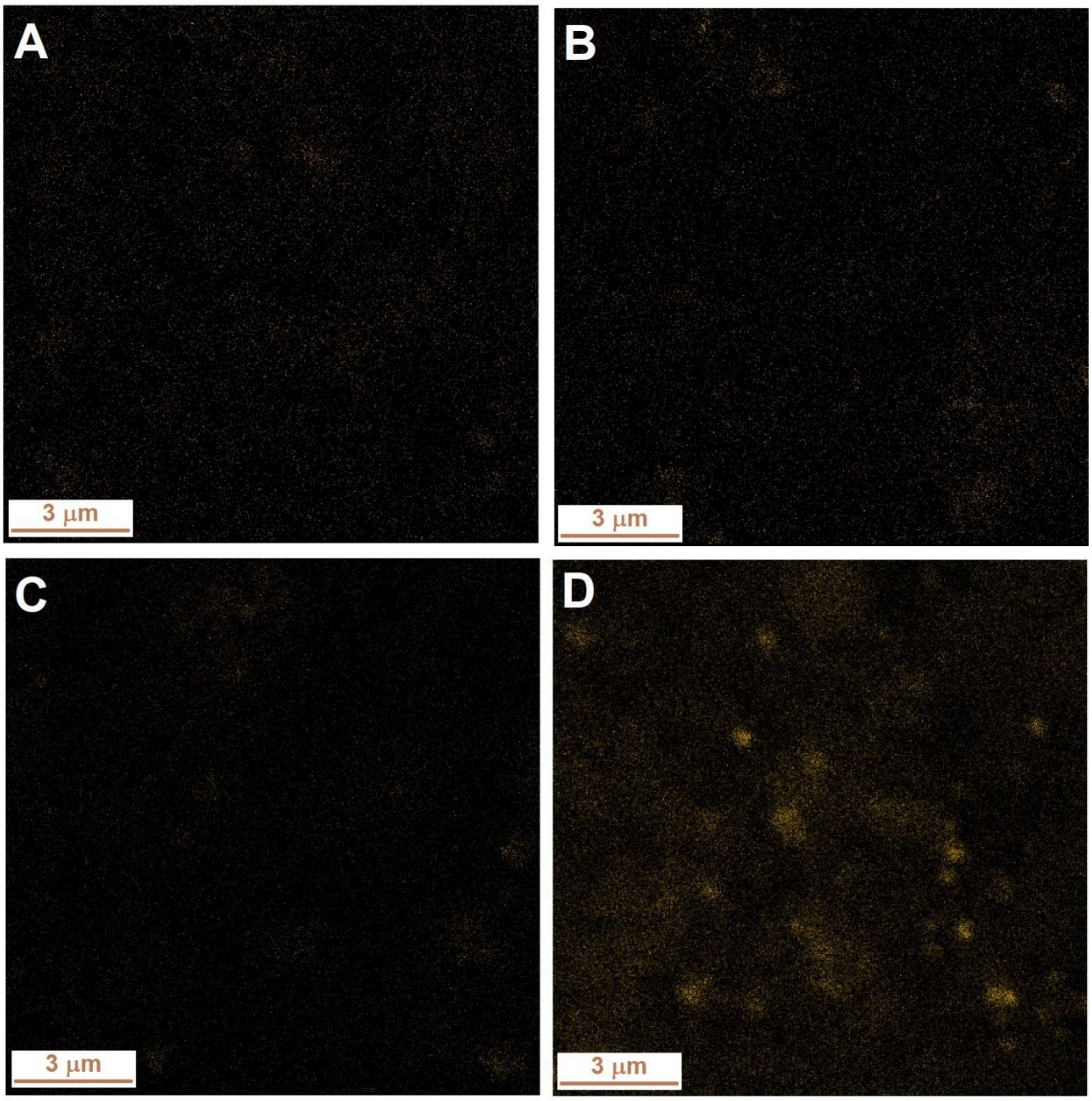


**Figure S7.** The influence of the duration of the ultrasonic-assisted Cu deposition on the appearance of Energy-dispersive X-ray spectroscopy (EDS) distribution maps over the surface of Cu-containing zeolite samples from the NaF31 series. **(A)** CuNaF31, **(B)** CuNaF31 (15), **(C)** CuNaF31 (30), and **(D)** CuNaF31 (60).

**Table S1.** Chemical composition of variously prepared Cu-F31 samples

| Sample | Cu concentration | | EDS chemical analysis | | Cu loading [%wt.] | Exchange level [%] | |
|---|---|---|---|---|---|---|---|
| | $Cu^{+}_{exch}$ [µmol/g] | $Cu^{+}_{oxide}$ [µmol/g] | Na [%wt.] | Cu [%wt.] | Cu | Cu | Na |
| **HF31** | n.a. | n.a. | n.a. | n.a. | n.a. | n.a. | n.a. |
| **CuHF31** | 89 | 37 | n.a. | 3.51 | 2 | 77 | n.a. |
| **CuHF31 (15)** | 85 | 24 | n.a. | 3.50 | 2 | n.d. | n.a. |
| **CuHF31 (30)** | 84 | 23 | n.a. | 3.49 | 2 | n.d. | n.a. |
| **CuHF31 (60)** | 66 | 19 | n.a. | 3.53 | 2 | 70 | n.a. |
| **NaF31** | n.a. | n.a. | 0.73 | n.a. | n.a. | n.a. | 91 |
| **CuNaF31** | 11 | 27 | 0.83 | 7.09 | 5 | n.a. | n.a. |
| **CuNaF31 (15)** | 10 | 19 | 0.82 | 5.60 | 5 | n.a. | n.a. |
| **CuNaF31 (30)** | 10 | 18 | 0.81 | 6.40 | 5 | n.a. | n.a. |
| **CuNaF31 (60)** | 6 | 11 | 0.92 | 6.22 | 5 | n.a. | n.a. |
